# A New Measurement Approach for Small Deformations of Soil Specimens Using Fiber Bragg Grating Sensors

**DOI:** 10.3390/s17051016

**Published:** 2017-05-04

**Authors:** Dong-Sheng Xu

**Affiliations:** School of Civil Engineering and Mechanics, Huazhong University of Science and Technology, 1037 Luoyu Rd., Wuhan 430074, China; dsxu@hust.edu.cn

**Keywords:** small deformation transducer, fiber Bragg grating (FBG), soil specimen, triaxial test

## Abstract

A measurement approach for small deformations of soil specimens has been proposed in this study. The proposed approach consists of a small deformation transducer (SDT) based on fiber Bragg grating sensors which could provide an alternative tool to measure local small deformations of a soil specimen with high accuracy. The working principle, design procedures, calibrations and applications of the SDT are presented. An analytical solution is derived to obtain the relationship between the small deformation of the transducer and the wavelength shift of the FBG sensor, which was further evident in the laboratory calibration tests. The measurement range and resolution of the SDT can be adjusted by choosing different length and thickness of the material. The SDT can achieve a strain resolution of 4.45 micro-strains for a soil specimen with 80 mm in height. Measurement errors and stability were also examined and the results show that the maximum measurement error was around 0.01 mm. The designed SDT was further installed in a modified triaxial apparatus. Three shearing tests under different confining pressures were conducted. Results measured by the newly developed SDT are analyzed with comparisons to the results using external linear variable differential transformer (LVDT) transducers. The results provide evidence that this measurement approach is suitable for measuring the local deformations of soil specimens with high accuracy and stability.

## 1. Introduction

Knowledge about the small deformation behavior of gravel materials is essential for the design and safety control of geotechnical structures. Many researchers have highlighted the importance of accurate measurements of the small strains of gravel material, such as soil specimens and aggregate [1,2,3]. In the past few decades, various methods and techniques have been developed to measure small strain behavior, such as bender elements, the resonant column method, proximity transducers, miniature linear variable differential transformer sensors (mini-LVDTs), mini-inclinometers, Hall-effect transducers, local deformation transducers (LDTs), etc. [4,5]. Among these, the bender element and LDT are the most commonly used methods to measure soil small strain behavior. The bender element is an effective method to measure very small strain behavior of soil specimens by using shear waves [6]. However, the soil strain induced by the shear wave can only be estimated, for example, Dyvik and Madshus [7] regarded the strain value is in the range of 10^−5^, while Leong et al. [8] and Pennington [9] estimated it around 10^−6^. To overcome the above limitations of the bender element method, the LDT was developed to measure local strains in the range between 10^−5^ and 10^−2^. The LDT not only can measure small soil strains with higher accuracy, but also measure continuous strains during the shearing phase. Thus, many miniature displacement devices were employed in the LDT for traxial test equipment (e.g., Jardine et al. [2], Clayton et al. [6], Hird and Yung [10], Goto et al. [11]). Goto et al. [11] were the first to develop a LDT with strain gauges to measure local small strains of soil specimens in a triaxial test machine. Later on, Dasari et al. [12] improved the accuracy with four strain gauges in one LDT and Yimsiri et al. [13] further developed a cantilever-based LDT with four strain gauges. However, the measurement accuracy by using strain gauge was limited due to the electrical and mechanical noises. Dasari et al. [12] pointed out the total noise can reach 6.0 mV at the beginning axial strain of 4 × 10^−5^ and then may increase with the increase of strain. Goto et al. [11] summarized eight sources of errors for LDTs, including electrical and mechanical noises from the system, hysteresis in calibration, changes of elastic behavior of the membrane and short circuit problems in the presence of high water pressure. Also, the self-weight of LDT devices will also reduce the measurement accuracy [14]. Thus, with the development of fiber optic sensors (FOS), it is necessary to develop a new measurement approach to measure local deformations of soil specimens with FOS.

In recent decades, FOS have been dramatically developed and widely applied in engineering practice [15,16,17,18,19]. Pei et al. [20] developed a FBG-based inclinometer and used it in field slope monitoring; Zhu et al. [21] applied FBG sensors to measure the internal displacement in a model dam. Compared with electrical strain gauges, the fiber optic sensors have obvious advantages, such as tiny size, high precision, resistance to electromagnetic interference, and a combination of sensing and transmission. Thus, in this study, a small deformation transducer (SDT) is developed to measure local deformation of soil specimens by using fiber Bragg grating (FBG) sensors. The aim of this study is to provide an effective approach to obtain the soil behavior under small deformation conditions, because soils adjacent to, for example, a subway tunnel, deep excavation foundations and retaining walls, is normally under small strain conditions. Thus, understanding soil small strain behavior is essential for civil engineers. The proposed approach could provide an effective tool to measure soil stress-strain behavior at small strain levels. In this paper, the design, working principle, fabrication, and calibration of the SDT are elaborated. A triaxial test apparatus was modified to employ the SDT. Several triaxial tests were conducted to evaluate the performance of the modified test system with the SDT.

## 2. Principle of the Small Deformation Transducer

### 2.1. Principles of the FBG Sensor

**Figure 1:**
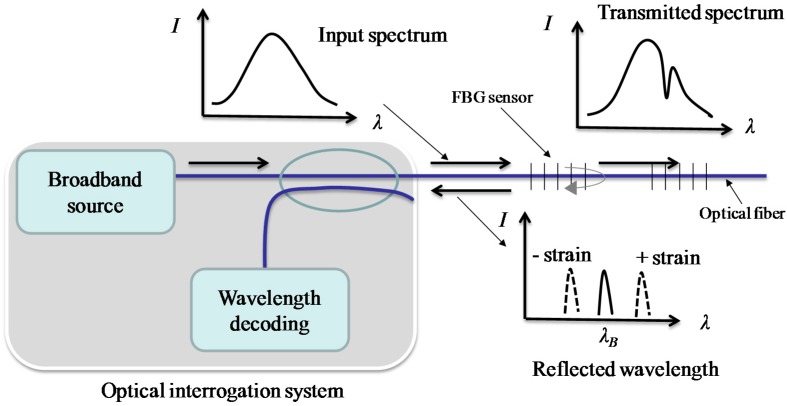
Illustration of the working principle of FBG sensors.

Figure 1 shows the working principle of the FBG sensor. As shown in the figure, when a broadband light source is passed through the FBG sensor, a narrow band light will be reflected. 

The reflected wavelength *λ*_B_ has a relationship with the core index of refraction and grating period of the index modulation, which is expressed as follows:

(1) $$\lambda_{B}=2n_{eff}\Lambda$$

where *n_eff_* is the core index of refraction and *Λ* is the grating period of index modulation. The reflected wavelength *λ_B_* will change with the local strains and/or temperatures. The wavelength change Δ*λ_B_* induced by strains and temperatures is given by [22]:

(2) $$\frac{\Delta \lambda_{B}}{\lambda_{B}}=\left[1-p_{e}\right]\cdot \varepsilon +\left(\alpha +\xi \right)\Delta T≅0.78\varepsilon +6.7\times 10^{-6}\Delta T$$

where *p_e_* is the elastic optical coefficient; *α* and *ξ* are temperature effect-related coefficients; Δ*T* is the temperature change.

**Figure 2:**
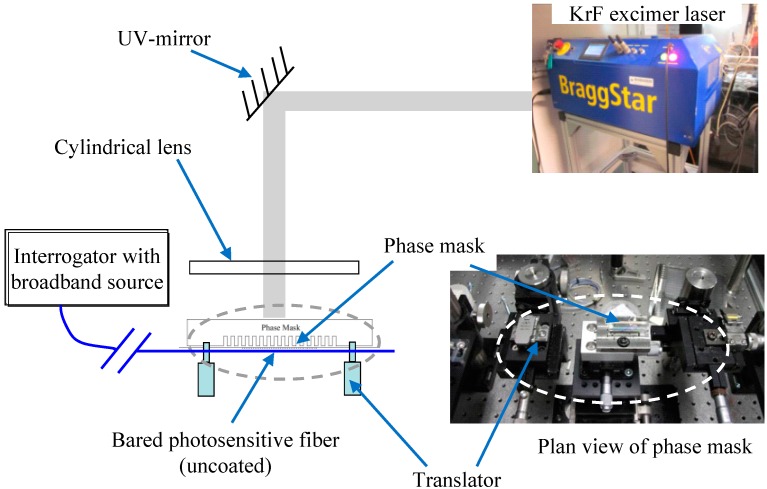
Experimental setup for fabricating FBG sensors by the phase mask method [23].

In this study, the FBG sensor was fabricated by the phase mask method. Detailed procedures for fabricating FBG sensors were described by Xu [23]. Figure 2 shows a sketch of the experimental setup used to fabricate FBG sensors by a phase mask method. A bare optical fiber was put in front of the phase mask which has a specified wavelength. After the fiber was exposed to a spatial pattern of ultraviolet light, a specific Bragg grating wavelength was written into the fiber. Briefly, the whole fabrication procedures can be divided into three stages: (a) preparation of the photosensitive optical fiber; (b) creating a Bragg grating using the phase mask method; and (c) thermal annealing [23].

### 2.2. Principle of the SDT

**Figure 3:**
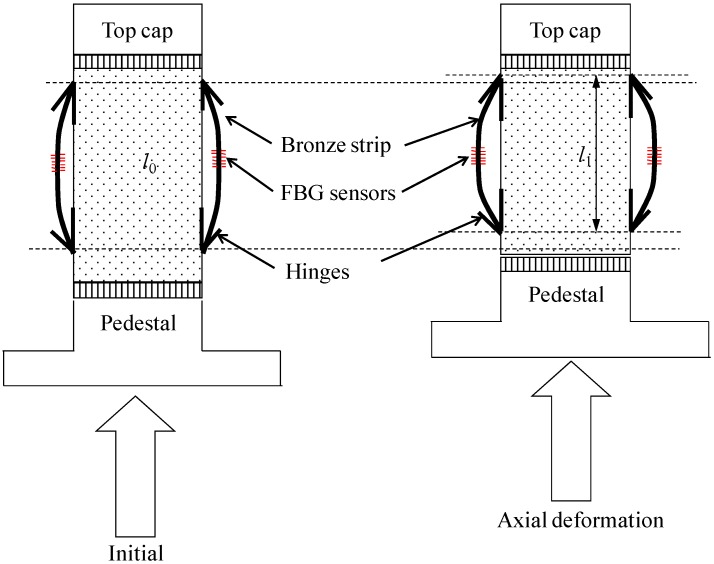
Schematic illustration of working principle of the SDT.

Figure 3 shows a diagram of the working principle of the designed SDT. The main components of the SDT include a flexible bronze strip, two FBG sensors and two hinged attachments. The two hinged attachments serve to fix the strip to the soil specimen. FBG sensors were glued at the centre of the bronze strip. Therefore, with the hinged attachment fixing device, the strip together with the FBG sensor can be deformed with the soil specimen.

**Figure 4:**
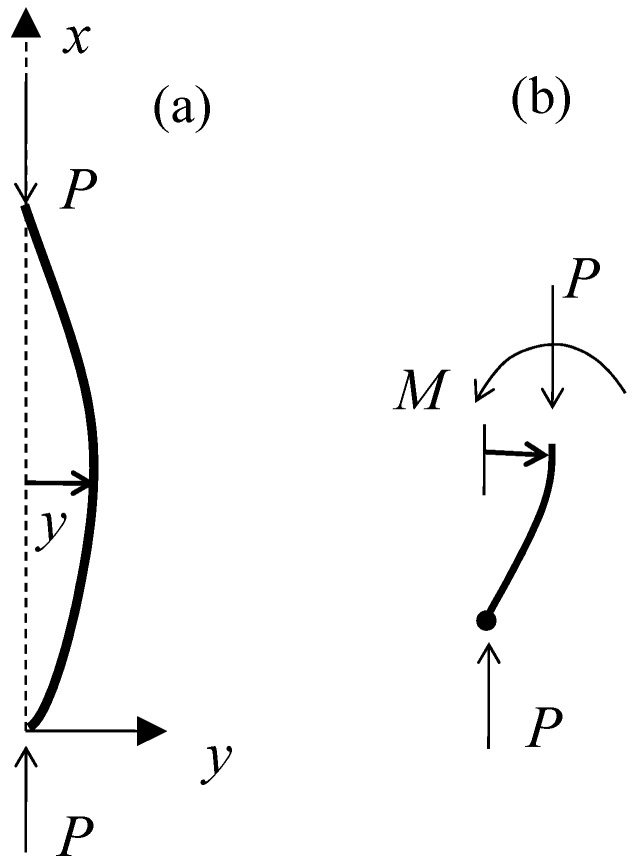
Working principle of the SDT: (**a**) deformed SDT; (**b**) force and moment balance within the SDT.

The working principle of the SDT is shown in Figure 4. According to the classic elastic equation of the beam, the deformation of the SDT can be determined with boundary conditions of *x* = 0, *y* = 0 and *x* = *l*, *y* = 0. The deformation of the SDT Δ can be expressed as:

(3) $$\begin{array}{l} \Delta =\int_{0}^{l}\sqrt{1+{\left(dy/dx\right)}^{2}}-l\\ \;\approx \int_{0}^{l}\left[1+\frac{1}{2}{\left(dy/dx\right)}^{2}\right]dx-l\\ \;=\frac{1}{2}\int_{0}^{l}{\left(dy/dx\right)}^{2}dx \end{array}$$

By considering the SDT deformed in the first model of bulking, the deflection curve *y* as indicated in Figure 4 is obtained as:

(4) $$y=c_{1}\sin\left(\pi x/l\right)$$

Substituting Equation (4) to (3), we can get:

(5) Δ=(c1π)2(4l) or c1=2lΔπ

The bending moment at the point *x*_0_ is expressed as:

(6) $$M_{0}=EI{\left(\pi /l\right)}^{2}c_{1}\sin\left(\pi x_{0}/l\right)$$

The tangential stress at *x*_0_ on the surface of the strip is:

(7) $$\sigma_{0}=M_{0}t/2I$$

where ‘*t*’ is the thickness of the strip in this study. Combining Equations (5)–(7) and together with Hook’s law $\sigma_{0}=E\varepsilon_{FBG}$, we have:

(8) $$\varepsilon_{FBG}=\sqrt{\frac{\Delta }{l}}\left(\frac{\pi t}{l}\right)\sin\left(\pi x_{0}/l\right)$$

If the FBG sensor is glued at *x*_0_ = *l*/2, so that:

(9) $$\frac{\Delta \lambda_{B}}{\lambda_{B}}=0.78\sqrt{\frac{\Delta }{l}}\left(\frac{\pi t}{l}\right)$$

where *t* and *l* are the thickness and length of the SDT, respectively. From Equation (9), it can be found that the axial deformation Δ has a unique relationship with the wavelength shifts of the FBG sensor. Therefore, the local axial deformations of the soil specimen can be obtained by the FBG sensor. The derived theoretical relationship between the measured wavelengths of FBG sensors and SDT deformations could provide a useful basis of analyzing the experimental calibration results. In addition, the theoretical analysis could help to design the SDT for various applications, such as adjusting the measurement accuracy and range though the parameter *t* and *l* for different conditions. It can also be obtain from the theoretical analysis that the sensitivity of the axial deformation Δ measured by the SDT can be increased by a larger thickness and shorter length of the SDT. From the assumptions of the theory, we can also understand that the SDT is limited to small deformation measurements as it was assumed to be deformed as a first model of bulking. However, the measurement range of deformation can be increased with larger length and a thin SDT material by considering Equation (9). It should be noted that if the FBG sensors are glued slightly off the center of the strip (i.e., *x*_0_ ≠ *l*/2), the Equation (9) is only approximate, then a laboratory calibration test is necessary to verify the relationship between the FBG sensor and the deformations. By considering the resolution of Δλ_B_ is 0.02 nm at the wavelength λ_B_ = 1550 nm according to the parameters of optical interrogation system used in this study (i.e., MOI SM130), the resolution of the SDT computed through Equation *(*9*)* is 3.55 × 10^−4^ mm, as the length and thickness of the SDT are 80 mm and 0.2 mm, respectively. Thus, if we used the SDT to measure a soil specimen with *a* height of 80 mm, the small strain resolution measured by the SDT is 4.45 micro-strains (με).

### 2.3. Fabrication of the SDT

The schematic diagram of the SDT is presented in Figure 3. There are four steps to prepare the SDT. Firstly, the FBG sensors with wavelength between 1510 nm and 1590 nm were prepared. Secondly, a spring-bronze strip was fabricated with 80 mm in length, 3.0 mm in width and 0.2 mm in thickness. The strip should be pre-strained with around 100 με to improve the linear properties and reduce creep behavior. Then, the FBG sensors were carefully installed on the surface of the bronze strip at the central position. It should be noted that the FBG sensors should be in good contact with the strip. Finally, the FBG sensors were protected by a thin layer of glue. All the sensors were connected in series and extended to the optical interrogator with an armed optical fiber.

### 2.4. Calibration of the SDT

**Figure 5:**
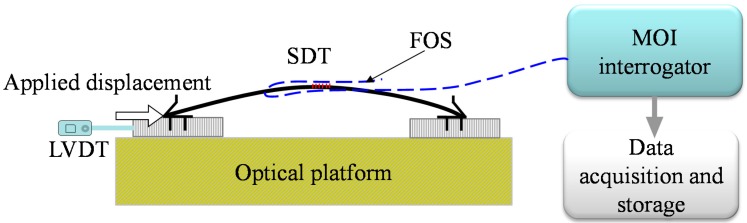
Setup of the calibration test.

The SDTs were calibrated in the laboratory with a constant temperature of 20 °C. Figure 5 shows the setup of the calibration test. The optical interrogation system with four optical channels, wavelength resolution of 0.02 nm, and the maximum scan frequency of 1 kHz used in this study was supplied by Micron Optics Incorporation (Atlanta, GA, USA). The interrogator is connected to a computer through an internet cable. As shown in the Figure 5, a special small scale screw was used to control the deformation of the SDT. The deformation was measured by a LVDT with a resolution of 1 × 10^−6^ m. The LVDT results were compared with the SDT. A separate temperature sensor can be used to measure the temperature and deduct any temperature effect if there are large temperature fluctuations during the test. In other words, temperature compensation can be conducted with a separate FBG temperature sensor.

**Figure 6:**
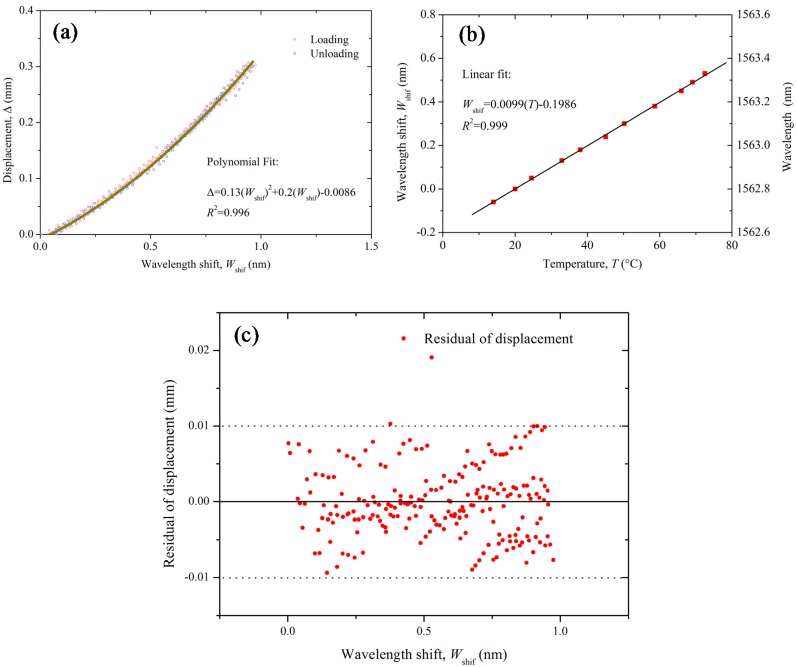
Calibration results of the SDT: (**a**) calibration results of SDT deformation versus wavelength shift of the FBG sensor; (**b**) calibration results of wavelength shifts versus temperatures changes; and (**c**) residual analysis of the polynomial fit in figure (**a**): residual of displacement versus wavelength shift, *W_shif_*.

Figure 6 shows the calibration results of the SDT with loading and unloading cycles and temperature effects. The wavelength shift (*W_shif_*) represents the measured wavelength minus the initial wavelength of the FBG sensors. Figure 6a shows the relationship between the deformations of the SDT and wavelength shift of the FBG sensor. The relationship is expressed as:

(10) $$\Delta =0.13{\left(W_{shif}\right)}^{2}+0.2\left(W_{shif}\right)-0.0086$$

where Δ denotes the deformation of the SDT; *W_shi_*_f_ is the wavelength shift of the FBG sensor. Based on the previous analysis, the deformation of the SDT has a quadratic function with the wavelength shift of the FBG sensor. Thus, a polynomial function was used to fit the relationship between deformation of the SDT and the wavelength shifts. Calibration results show that the fitting accuracy is quite high (*R*^2^ = 0.996). A little initial hysteresis was observed in this calibration, thus it is suggested to apply an initial bending when setting up the SDT which can reduce the initial hysteresis. The temperature effects on the wavelength shifts *(W_shif_*) of the SDT was calibrated in the laboratory and presented in Figure 6b. It shows that the wavelength of the FBG sensor has a close linear relationship with the surrounding temperatures. If we take the wavelength of temperature sensor at 20 °C as the initial value, the relationship between the wavelength shift *W_shif_* (i.e., FBG wavelength minus FBG wavelength at 20 °C) and the temperature can be expressed as:

(11) $$W_{shif}=0.0099\left(T\right)-0.1986$$

Thus, the wavelength shift induced by the temperature variations can be deducted by Equation (11) from the measured results of SDT. Figure 6c shows the residual analysis of polynomial fit in Figure 6a. The residual displacement of the SDT calculated by Equation (10) minus the measured displacement by LVDT shows the maximum measurement error is around 0.01 mm. From the calibration results, it can be found that the overall accuracy of the SDT is quite high. However, it should be noted that the sensitivity of the SDT will be decreased if the deformation is too large. Large deformation will induce higher contact force between the membrane and the hinge which will also diminish the accuracy. Therefore, it is suggested to keep the maximum displacement of the SDT below 0.3 mm.

### 2.5. Stability and Error Analysis of the SDT

**Figure 7:**
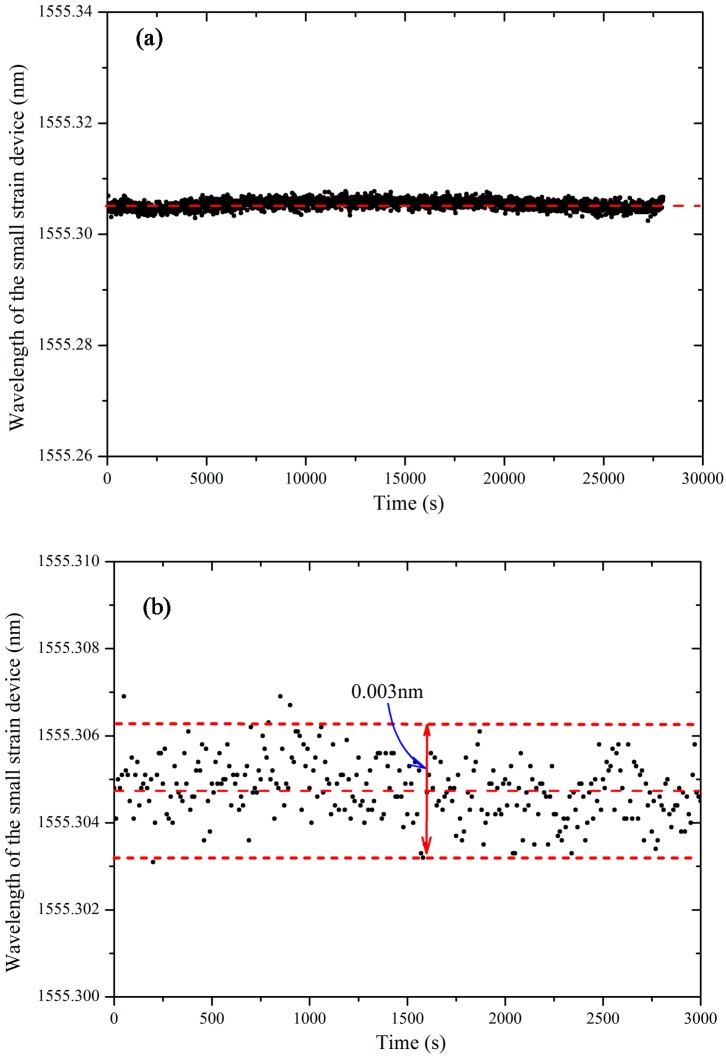
Performance examination of the designed SDT: (**a**) long-term measured wavelengths; (**b**) measured errors of the wavelength.

The stability of the SDT is a significant concern for deformation measurement. A laboratory stability test of the SDT was conducted for a relatively long-time of 30,000 s. Figure 7a shows the directly measured wavelength results of the SDTs. It shows that the measured wavelengths were very stable during 30,000 s.

The measurement errors of the SDT come from two sources: the first is the calculation error based on Equation (10) and the second source is the measurement error of the FBG sensor. Based on the Equation (10), the standard measurement error from the SDT can be expressed as:

(12) $$\sigma_{\Delta }=\frac{\partial \left(\Delta \right)}{\partial \left(W_{shif}\right)}\cdot \sigma \left(W_{shif}\right)$$

where *σ*_Δ_ is the standard error of the SDT; *σ*(*W_shif_*) is the error of wavelength of the FBG sensor.

The directly measured error from the FBG sensor was examined in this study. The SDT was installed in a soil specimen and continuous readings were taken under static condition. Figure 7b shows the taken readings of the FBG sensors under static condition. It can be found that the *σ*(*W_shif_*) is equal to ±0.0015 nm. Substituting Equation (10) into (11) the results of *σ*(*W_shif_*), the standard error of the SDT can be obtained as:

(13) $$\sigma_{\Delta }=\frac{\partial \Delta }{\partial \left(W_{shif}\right)}\sigma \left(W_{shif}\right)=\pm 0.0015\left(0.26W_{shif}+0.2\right)$$

From Equation (13), as the maximum wavelength shift of the FBG sensor is 3 nm, the maximum error of the measured results by the SDT is ±0.00147 mm.

## 3. Experimental Setup

### 3.1. A Modified Test Apparatus with SDT

**Figure 8:**
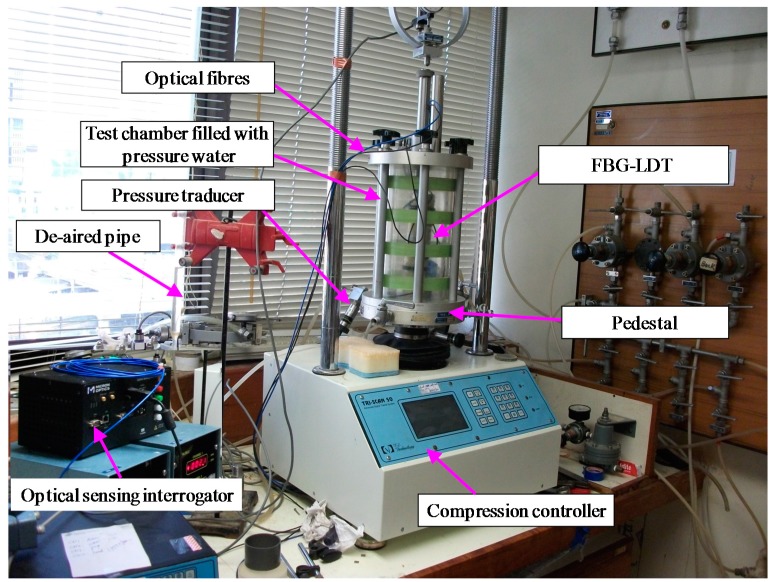
Schematic view of SDT test system in the modified testing apparatus.

**Figure 9:**
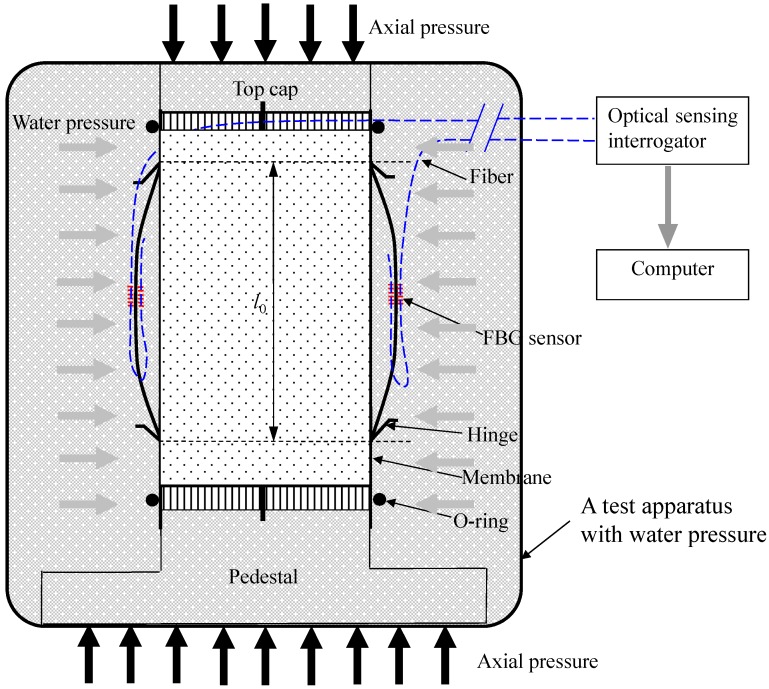
Schematic diagram of the SDTs on a soil specimen in a modified traxial apparatus.

A modified traditional triaxial apparatus was used to install the SDT. The schematic setup of the test apparatus is shown in Figure 8. The SDT was calibrated and then fixed on the surface of the soil specimen by two hinges. As mentioned early, an initial bending should be applied so that the distance between the two hinges was kept 1 mm shorter than the length of the SDT. It should be noted that the initial bending depends on the cyclic test settings, because the SDT will experience both extension and compression during cyclic tests. Two SDTs were mounted on the surface of the specimen in diametrically opposite to each other. The test system includes a triaxial test chamber with a loading frame, pressure supply devices, the SDT, fiber optic sensing interrogator and computer. In this study, the external LVDT device was also used to measure the total deformation of the soil specimen. The SDT was used to measure the local deformation with the maximum strain up to 0.3%. Three different tests were conducted with different confining pressure. After the soil specimen and the SDT were installed, the chamber was mounted and filled with distilled water. Distilled water will be used to apply surrounding pressures to the soil specimen as indicated in Figure 9. A loading rate of 0.005 mm/min was applied to shear a completely decomposed granite (CDG) soil specimen under a constant confining pressure. During shearing process, both the SDT and the LVDT data were recorded.

### 3.2. Properties of the Soil Specimen

The SDT was tested in a modified traxial apparatus for a completely decomposed granite (CDG) soil. The tests were conducted by following guidelines of BS 1337-2, 1990; BS-1337, 1990; BS-5930, 1999. The liquid limit (*w_L_*), plastic limit (*w_p_*), plasticity index (*I*_p_) and the specific gravity (*G_s_*) were 31%, 21%, 10%, and 2.59, respectively. The soil was classified as silty sand according to the USCS classification (ASTM D 2487). The maximum dry density (MDD) and optimum moisture content (OMC) of the soil were found to be 1.84 Mg/m^3^ and 13.5%, respectively, based on standard compaction tests.

### 3.3. Test Procedures

The soil specimens were prepared by pretreating disturbed soil, mixing with water and compacting to a target density. The soil was over dried by keeping in the oven for three days under a constant temperature of 104 °C. The over dried soil was then mixed with distilled water to achieve an optimum moisture content of 13.5%. To ensure even water content, the soil was matured for 24 h at a constant room temperature of 20 °C. The prepared soil was then compacted in three layers in a mold with dimension of 50 mm in diameter and 100 mm in height to achieve a dry density of 1.75 Mg/m^3^ (i.e., 95% of the maximum dry density of 1.84 Mg/m^3^).

The specimen was properly mounted on a modified triaxial apparatus and sealed in a latex rubber membrane with O-rings. Two SDTs were installed on the surface of the specimen as shown in Figure 9. Each SDT was attached on the membrane by employing two metal hinges which were glued on the membrane with a special epoxy resin. After the specimen and SDTs were installed, the outer chamber was filled with distilled water. Then, the specimen was saturated under a confining pressure of 50 kPa and back pressure of 45 kPa. After completion of saturation, the specimen was consolidated with a target isotropic confining pressure. The consolidation is finished when the degree of consolidation is found to be 95%. The specimen was then sheared with a shearing rate of 0.005 mm/min under a constant confining pressure. During shearing, the readings of the SDTs and external LVDTs were continuously recorded by the data logger and a computer.

## 4. Test Results and Discussions

**Figure 10:**
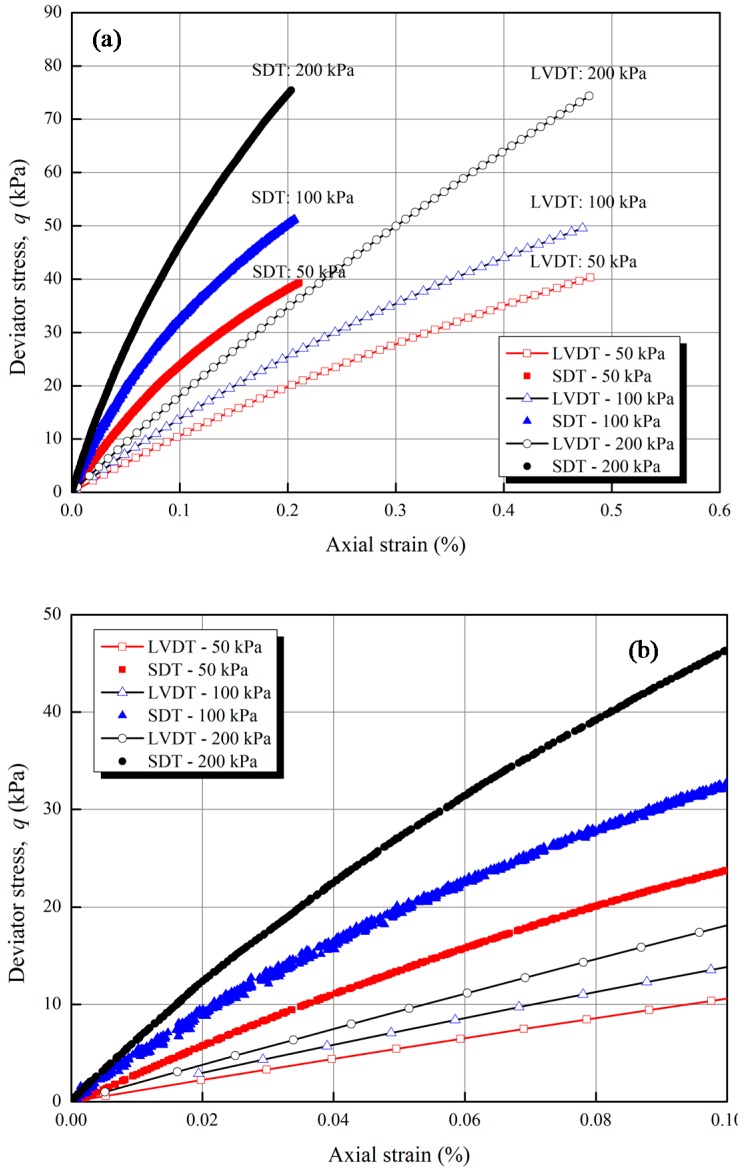
Stress-strain relationship between deviator stress and axial strain under different confining pressures: (**a**) Strain scale of 0 to 1%; (**b**) Strain scale of 0 to 0.1%.

Figure 10 shows three stress-strain results of the SDTs and the external LVDTs under constant confining pressures of 50 kPa, 100 kPa, and 200 kPa. In order to facilitates a detailed performance evaluation of the SDT, the results of stress-strain relationship are plotted using different maximum scales (i.e., 0.1% and 0.5%) of axial strain (as depicted in Figure 10a,b). From Figure 10b, the external LVDT cannot measure the strain below 0.01% because of the bending errors and system compliances. It is observed that the local SDT results are appreciably smaller than the external LVDT measurements. The same findings from literature [2,6,11] indicated that the external LVDT overestimates the axial strains because of the bending errors and system compliances. Goto et al. [11] found that the axial strain measured by external device was almost twice higher than the strain measured by local deformation transducer. As the SDT is proposed to measure local deformations of soil specimen as indicated in Figure 9, the current LVDTs cannot be directly installed at the same positions of the SDT in the soil specimen due to the limited space of the triaxial apparatus and the specimen. The independent comparison of the SDT was conducted in the calibration tests as elaborated in the previous sections. Here, the SDT was only compared with external LVDTs which were also reported in the literature [6,11].

The accuracy of the SDT depends on several factors, such as: (1) the resolution of the FBG sensors; (2) the resolution of the fiber optic sensing interrogator; (3) the calibration coefficient; (4) the glue quality of the FBG sensor and the hinges; (5) any creep errors introduced between the membrane and the soil specimen. The items (1) and (2) are sources of errors common in any FBG sensor and also in traditional electrical transducers. The initial hysteresis is negligible and can be reduced by applying initial bending when setup the SDT during the test. The contact force between hinges and membrane increases with the deformation of SDT, which may further lead to the creep in hinges. However, the creeps of the hinges were examined by Goto et al. [11]. The results indicated that the rate of creep of the hinges introduced between the membrane and the soil specimen was 1 µm per day. Thus, considering the testing period for a standard soil test, the creep effect is negligible. Considering the described factors, it can be concluded that the SDT can be used effectively to measure the small strain behavior of soil specimens with good accuracy.

## 5. Conclusions

In this study, a measurement approach for small deformations of soil specimens was proposed. A small deformation transducer (SDT) has been designed, fabricated, calibrated and installed in a modified triaxial apparatus. An analytical solution has been developed to obtain the relationship between the wavelength shifts of the FBG sensors and the local deformations of the SDT. The calibration test of the SDT proves that it can be used to measure local deformations and enables it to determine soil strength and stiffness at small strain levels. The SDT was employed in a modified triaxial test for measuring the small deformation behavior of soil specimens under different confining pressures. Different confining pressure tests were conducted and the results prove that the SDT works well in the presence of water. The SDT is more feasible to be used for local deformation measurements of soil specimens in a triaxial apparatus as it has obvious merits such as light weight, high accuracy, resistance to corrosion and ease of handling. It can be hoped that the SDT can be further developed for investigating the small deformation behavior of other materials such as rock and concrete under different loading conditions.
